# Progressive cerebellar atrophy in a patient with complex II and III deficiency and a novel deleterious variant in SDHA: A Counseling Conundrum

**DOI:** 10.1002/mgg3.1692

**Published:** 2021-05-07

**Authors:** Beattie R. H. Sturrock, Ellen F. Macnamara, Peter McGuire, Shannon Kruk, Ivan Yang, Jennifer Murphy, Undiagnosed Diseases Network, Maria T Acosta, Margaret Adam, David R Adams, Pankaj B Agrawal, Mercedes E Alejandro, Justin Alvey, Laura Amendola, Ashley Andrews, Euan A Ashley, Mahshid S Azamian, Carlos A Bacino, Guney Bademci, Eva Baker, Ashok Balasubramanyam, Dustin Baldridge, Jim Bale, Michael Bamshad, Deborah Barbouth, Pinar Bayrak‐Toydemir, Anita Beck, Alan H Beggs, Edward Behrens, Gill Bejerano, Jimmy Bennet, Beverly Berg‐Rood, Jonathan A Bernstein, Gerard T Berry, Anna Bican, Stephanie Bivona, Elizabeth Blue, John Bohnsack, Carsten Bonnenmann, Devon Bonner, Lorenzo Botto, Brenna Boyd, Lauren C Briere, Elly Brokamp, Gabrielle Brown, Elizabeth A Burke, Lindsay C Burrage, Manish J Butte, Peter Byers, William E Byrd, John Carey, Olveen Carrasquillo, Ta Chen Peter Chang, Sirisak Chanprasert, Hsiao‐Tuan Chao, Gary D Clark, Terra R Coakley, Laurel A Cobban, Joy D Cogan, Matthew Coggins, F Sessions Cole, Heather A Colley, Cynthia M Cooper, Heidi Cope, William J Craigen, Andrew B Crouse, Michael Cunningham, Precilla D'Souza, Hongzheng Dai, Surendra Dasari, Mariska Davids, Jyoti G Dayal, Matthew Deardorff, Esteban C Dell'Angelica, Shweta U Dhar, Katrina Dipple, Daniel Doherty, Naghmeh Dorrani, Emilie D Douine, David D Draper, Laura Duncan, Dawn Earl, David J Eckstein, Lisa T Emrick, Christine M Eng, Cecilia Esteves, Tyra Estwick, Marni Falk, Liliana Fernandez, Carlos Ferreira, Elizabeth L Fieg, Laurie C Findley, Paul G Fisher, Brent L Fogel, Irman Forghani, Laure Fresard, William A Gahl, Ian Glass, Rena A Godfrey, Katie Golden‐Grant, Alica M Goldman, David B Goldstein, Alana Grajewski, Catherine A Groden, Andrea L Gropman, Irma Gutierrez, Sihoun Hahn, Rizwan Hamid, Neil A Hanchard, Kelly Hassey, Nichole Hayes, Frances High, Anne Hing, Fuki M Hisama, Ingrid A Holm, Jason Hom, Martha Horike‐Pyne, Alden Huang, Yong Huang, Rosario Isasi, Fariha Jamal, Gail P Jarvik, Jeffrey Jarvik, Suman Jayadev, Jean M Johnston, Lefkothea Karaviti, Emily G Kelley, Jennifer Kennedy, Dana Kiley, Isaac S Kohane, Jennefer N Kohler, Deborah Krakow, Donna M Krasnewich, Elijah Kravets, Susan Korrick, Mary Koziura, Joel B Krier, Seema R Lalani, Byron Lam, Christina Lam, Brendan C Lanpher, Ian R Lanza, C. Christopher Lau, Kimberly LeBlanc, Brendan H Lee, Hane Lee, Roy Levitt, Richard A Lewis, Sharyn A Lincoln, Pengfei Liu, Xue Zhong Liu, Nicola Longo, Sandra K Loo, Joseph Loscalzo, Richard L Maas, Ellen F. Macnamara, Calum A MacRae, Valerie V Maduro, Marta M Majcherska, Bryan Mak, May Christine V. Malicdan, Laura A Mamounas, Teri A Manolio, Rong Mao, Kenneth Maravilla, Thomas C Markello, Ronit Marom, Gabor Marth, Beth A Martin, Martin G Martin, Julian A Martínez‐Agosto, Shruti Marwaha, Jacob McCauley, Allyn McConkie‐Rosell, Colleen E McCormack, Alexa T McCray, Elisabeth McGee, Heather Mefford, J. Lawrence Merritt, Matthew Might, Ghayda Mirzaa, Eva Morava, Paolo M Moretti, Marie Morimoto, John J Mulvihill, David R Murdock, Mariko Nakano‐Okuno, Avi Nath, Stan F Nelson, John H Newman, Sarah K Nicholas, Deborah Nickerson, Shirley Nieves‐Rodriguez, Donna Novacic, Devin Oglesbee, James P Orengo, Laura Pace, Stephen Pak, J. Carl Pallais, Christina G. S. Palmer, Jeanette C Papp, Neil H Parker, John A. Phillips, Jennifer E Posey, Lorraine Potocki, Barbara N Pusey, Aaron Quinlan, Wendy Raskind, Archana N Raja, Deepak A Rao, Genecee Renteria, Chloe M Reuter, Lynette Rives, Amy K Robertson, Lance H Rodan, Jill A Rosenfeld, Natalie Rosenwasser, Maura Ruzhnikov, Ralph Sacco, Jacinda B Sampson, Susan L Samson, Mario Saporta, C. Ron Scott, Judy Schaechter, Timothy Schedl, Kelly Schoch, Daryl A Scott, Prashant Sharma, Vandana Shashi, Jimann Shin, Rebecca Signer, Catherine H Sillari, Edwin K Silverman, Janet S Sinsheimer, Kathy Sisco, Edward C Smith, Kevin S Smith, Emily Solem, Lilianna Solnica‐Krezel, Rebecca C Spillmann, Joan M Stoler, Nicholas Stong, Jennifer A Sullivan, Kathleen Sullivan, Angela Sun, Shirley Sutton, David A Sweetser, Virginia Sybert, Holly K Tabor, Cecelia P Tamburro, Queenie K.‐G. Tan, Mustafa Tekin, Fred Telischi, Willa Thorson, Cynthia J Tifft, Camilo Toro, Alyssa A Tran, Brianna M Tucker, Tiina K Urv, Adeline Vanderver, Matt Velinder, Dave Viskochil, Tiphanie P Vogel, Colleen E Wahl, Stephanie Wallace, Nicole M Walley, Chris A Walsh, Melissa Walker, Jennifer Wambach, Jijun Wan, Lee‐kai Wang, Michael F Wangler, Patricia A Ward, Daniel Wegner, Mark Wener, Tara Wenger, Katherine Wesseling Perry, Monte Westerfield, Matthew T Wheeler, Jordan Whitlock, Lynne A Wolfe, Jeremy D Woods, Shinya Yamamoto, John Yang, Guoyun Yu, Diane B Zastrow, Chunli Zhao, Stephan Zuchner, Cyndi J. Tifft, Eliza Gordon‐Lipkin

**Affiliations:** ^1^ National Institutes of Health Undiagnosed Diseases Program Common Fund Office of the Director NIH Bethesda MD USA; ^2^ Brighton and Sussex University Hospitals NHS Trust Brighton England; ^3^ Metabolism, Infection and Immunity Section National Human Genome Research Institute NIH Bethesda MD USA; ^4^ Common Fund Office of the Director NIH Bethesda MD USA; ^5^ Office of the Clinical Director National Human Genome Research Institute NIH Bethesda MD USA

**Keywords:** cerebellar atrophy, complex II, mitochondrial disease, novel mutation, SHDA

## Abstract

**Background:**

Complex II is an essential component of the electron transport chain, linking it with the tricarboxylic acid cycle. Its four subunits are encoded in the nuclear genome, and deleterious variants in these genes, including *SDHA* (OMIM 600857), are associated with a wide range of symptoms including neurological disease, cardiomyopathy, and neoplasia (paraganglioma‐pheochromocytomas (PGL/PCC), and gastrointestinal stromal tumors). Deleterious variants of *SDHA* are most frequently associated with Leigh and Leigh‐like syndromes.

**Methods and Results:**

Here, we describe a case of a 9‐year‐old boy with tremor, nystagmus, hypotonia, developmental delay, significant ataxia, and progressive cerebellar atrophy. He was found to have biallelic variants in *SDHA*, a known pathogenic variant (c.91C>T (p.R31*)), and a variant of unknown significance (c.454G>A (p.E152K)). Deficient activity of complexes II and III was detected in fibroblasts from the patient consistent with a diagnosis of a respiratory chain disorder.

**Conclusion:**

We, therefore, consider whether c.454G>A (p.E152K) is, indeed, a pathogenic variant, and what implications it has for family members who carry the same variant.

## Graphical abstract

Progressive cerebellar atrophy in a patient with complexes II and III deficiency and SDHA compound heterozygous variants. These variants were considered pathogenic, given respiratory chain findings, and the family was counseled based on the presence of pathogenic variants.

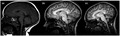

## 1 INTRODUCTION

Mitochondrial diseases comprise a heterogeneous group of inborn errors of metabolism resulting from deleterious variants in genes involved in oxidative phosphorylation and mitochondrial maintenance, which are encoded by either nuclear or mitochondrial DNA. The mitochondrial proteome contains 1100–1400 distinct proteins, most of which are encoded within the nuclear genome; only 13 proteins are encoded within the mitochondrial genome (Gorman et al., 2016). The key metabolic pathways within the mitochondria include the tricarboxylic acid cycle (TCA) and oxidative phosphorylation via the electron transport chain (ETC). Succinate dehydrogenase, also known as complex II, is a tetrameric mitochondrial protein complex required for both processes. Complex II, the second of four protein complexes in the ETC, links the TCA cycle to oxidative phosphorylation (Van Vranken et al., 2015). Unlike other complexes of the ETC, all of the succinate dehydrogenase subunits (SDHA, SDHB, SDHC, and SDHD) are encoded by the nuclear genome (Van Vranken et al., 2015). Mutations in all of these genes have been implicated in human diseases, including neoplasms (Aldera & Govender, 2018; Branca et al., 2017; Hoekstra & Bayley, 2013). Interestingly, pathogenic variants in *SDHA* have been implicated in cancer (Aldera & Govender, 2018; Hoekstra & Bayley, 2013) and mitochondrial disease. The genotypes and phenotypes associated with *SDHA*‐related mitochondrial disease are variable, with both dominant and recessive inheritance being reported (Baysal et al., 2001). Pathogenic variants produce variable phenotypes including Leigh syndrome (Horvath et al., 2006; Pagnamenta et al., 2006; Parfait et al., 2000; Renkema et al., 2015), bilateral optic atrophy (Birch‐Machin et al., 2000; Courage et al., 2017; Taylor et al., 1996), and cardiomyopathy (Courage et al., 2017; Levitas et al., 2010).

In this case report, we present a child who was found to have biallelic variants in *SDHA*, a known pathogenic variant and a variant of unknown significance. We consider the implications that this clinical picture has for the phenotypic spectrum of this disease and for the genetic counseling as it relates to the health risks of family members who carry the same variants.

## 2 CASE HISTORY AND METHODS

### 2.1 Ethical compliance

The proband was evaluated at the National Institutes of Health (NIH) under protocols 15‐HG‐0130, The Undiagnosed Diseases Network, and 13‐HG‐0053, The MINI Study (Metabolism, Infection and Immunity in Inborn Errors of Mitochondrial Metabolism), approved by the National Human Genome Research Institute Institutional Review Board.

The proband is the product of a full‐term pregnancy, with decreased fetal activity, to a non‐consanguineous 28‐year‐old G3P2AB1 mother and 40‐year‐old father. The proband was born at 40 weeks’ gestation and was noted to have a weak cry. His birth weight was 4.1 kg and length was 57.2 cm, resulting in weight for length ratio that was below the fifth percentile.

Neurological concerns began at 2 years of age when he presented with global developmental delays. He had impairments in both receptive and expressive language (developmental quotient of 70) and delayed walking at 19 months with a wide‐based, ataxic gait. There were no episodes of developmental regression. Despite physical therapy, he continued to have ataxia and frequent falls with foot drop noted at 3 years of age. At that time, he was also noted to have hypotonia, a coarse tremor, tongue fasciculations, end gaze nystagmus, and cogwheel visual pursuits. At 5 years of age, he had worsening ataxia and speech apraxia despite normal strength on neurological exam and a normal audiologic exam. A neuropsychological evaluation diagnosed intellectual disability with prominent deficits in processing speed and auditory processing.

**FIGURE 1:**
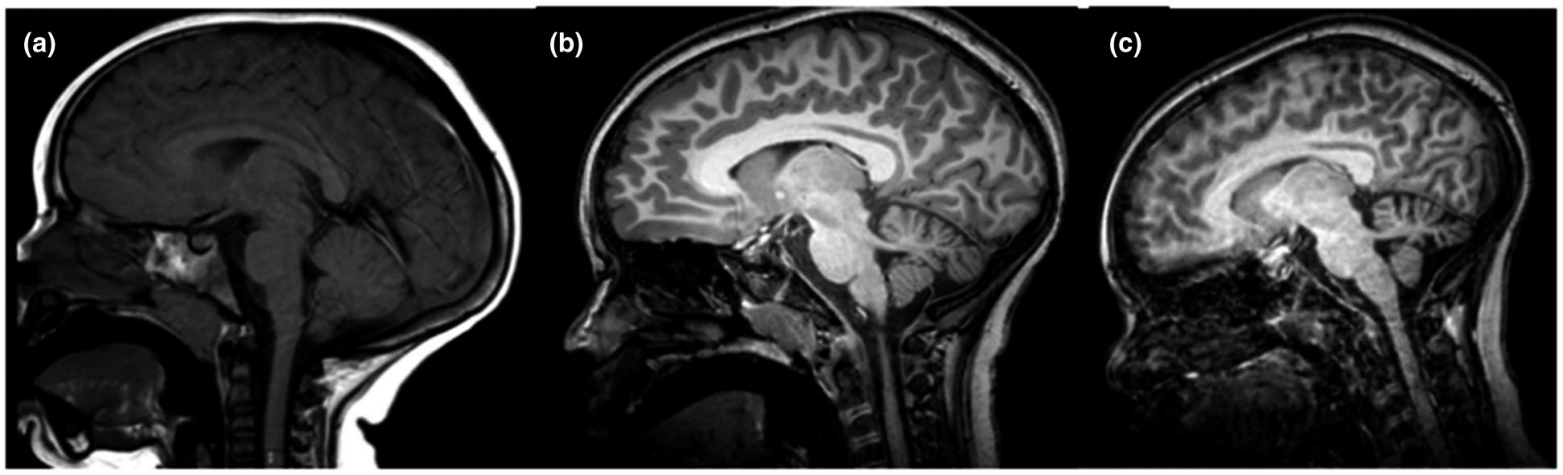
Serial brain MRIs illustrating progressive cerebellar atrophy at (a) age 2 years, (b) age 6 years, and (c) age 9 years

Neuroimaging, including magnetic resonance imaging (MRI) at 2 years of age and magnetic resonance angiogram (MRA) at 4 years of age, was normal. At 6 and 9 years of age, he had brain MRIs performed at the NIH, which suggested progressive cerebellar atrophy (Figure 1). MR spectroscopy at 6 years of age showed a deficit of *N*‐acetylaspartate in the superior cerebellar vermis and the pons, indicating the possibility that these regions are less neuron dense (Verma et al., 2016). Electromyography at 6 years of age was normal.

On examination at the NIH at 9 years of age, interpupillary distance, intercanthal distance, outer canthal distance, and palpebral fissure length were all >97th percentile; however, these were in proportion to his macrocephaly (head circumference >97th percentile). Growth parameters, at this time, include height and weight (>97% centile) with a resulting BMI of 26 kg/m^2^.

Family history was significant for multiple relatives with diabetes mellitus type II, high blood pressure, and heart disease. Except one paternal aunt with breast cancer, there was no known family history of cancer.

### 2.2 Genetic evaluation

Genetic testing was non‐diagnostic including Fragile X PCR, Prader–Willi/Angelman syndrome DNA methylation, *PTEN* gene sequencing, and chromosomal microarray analysis. Lysosomal enzyme screening of whole blood showed mildly reduced activities of beta‐galactosidase and beta‐glucuronidase. He had normal cerebral spinal fluid (CSF) glucose, lactate, amino acids, neurotransmitter metabolites, tetrahydrobiopterin, neopterin, and 5‐methyltetrahydrofolate levels. Mitochondrial genome sequencing was normal.

Whole‐exome sequencing of the proband and both unaffected parents, performed as part of the NIH UDP evaluation, identified compound heterozygous variants in *SDHA* with a maternally inherited, c.91C>T (p.R31*), known pathogenic variant and a paternally inherited, c.454G>A (p.E152K), variant of unknown significance (VUS). The variant c.91C>T (p.R31*) is present in gnomAD at a frequency of 0.0002088 and is assessed as pathogenic using ACMG criteria. The variant c.454G>A (p.E152K) is present in gnomAD at a frequency of 0.000007998 and is predicted to be damaging/probably damaging by SIFT/PolyPhen‐2, respectively. A maternally inherited hemizygous VUS in *SLC6A8* was also identified and ruled out as causative given normal blood and CSF creatine studies.

### 2.3 Respiratory chain functional studies

An analysis of the proband's mitochondrial respiratory chain enzyme function using skin fibroblasts showed modestly reduced citrate synthase activity and a deficiency in complexes II and III activity (<40%), fulfilling a minor criterion for the diagnosis of a respiratory chain disorder.

## 3 DISCUSSION

We describe a child with compound heterozygous deleterious variants in *SDHA* causing reduced complexes II and III activity resulting in moderate intellectual disability and progressive cerebellar ataxia. Our case is important in highlighting the novel variant, c.454G>A (p.E152K), previously classified as a variant of unknown significance, as likely pathogenic.

The spectrum of clinical phenotypes associated with *SDHA* mutations is broad (Table 1). Cases with dominant inheritance include one family with bilateral optic atrophy, ocular movement disorder, neuropathy, mental health conditions, and cardiomyopathy (Courage et al., 2017), and another family with neurodegeneration and bilateral optic atrophy (Birch‐Machin et al., 2000; Taylor et al., 1996). Homozygous or compound heterozygous *SDHA* variants have been found in patients with Leigh syndrome (Bourgeron et al., 1995; Horvath et al., 2006; Pagnamenta et al., 2006; Parfait et al., 2000; Renkema et al., 2015), leukodystrophy (Alston et al., 2012; Renkema et al., 2015), cardiomyopathy (Levitas et al., 2010), and progressive neuromuscular decline (Ma et al., 2014). While cerebellar signs have been reported in monoallelic *SDHA* variant cases (Birch‐Machin et al., 2000; Courage et al., 2017; Taylor et al., 1996), the childhood‐onset cerebellar atrophy described here is newly reported. Prior cases describe ataxia onset in adulthood (Birch‐Machin et al., 2000; Taylor et al., 1996).

**TABLE 1:** reported cases associated with deleterious variants in *SDHA*

Publication details	Age at presentation	SDHA variant(s)	Phenotype
Courage et al. (2017)	15 years	Heterozygous c.1351C>T (p.Arg451Cys)	Ocular paresis, nystagmus, pyramidal signs, ataxia, cardiomyopathy with cardiomegaly, recurrent depression
	8 months	Heterozygous c.1351C>T (p.Arg451Cys)	Dilated cardiomyopathy, bilateral optic atrophy, elevated urinary 3‐methylglutaconic and 3OH‐ methylglutaric excretion
	7 months	Heterozygous c.1351C>T (p.Arg451Cys)	Deceased due to cardiac insufficiency with dilated cardiomyopathy, marginally elevated blood lactate, elevated urinary 3‐methylglutaconic and 3OH‐methylglutaconic excretion
Taylor et al. (1996) Birch‐Machin et al. (2000)	46 years	Heterozygous c.1375C>T (p.Arg408Cys)	Ataxia, diplopia, limb weakness, episodic unresponsiveness without convulsions, bilateral optic atrophy
	62 years	Heterozygous c.1375C>T (p.Arg408Cys)	Ataxia, diplopia, blackouts, dysesthesia, bilateral optic atrophy, nystagmus on lateral and upward gaze
Bourgeois et al. (1992) Bourgeron et al. (1995)	10 months	Homozygous c.1684C>T (p.Arg544Trp)	Leigh syndrome
	10 months	Homozygous c.1684C>T (p.Arg544Trp)	Leigh syndrome
Renkema et al. (2015)	Birth	Compound heterozygous c.356G>A (p.Try119*) and c.248C>T (p.Ala83Gln104del)	Developmental regression, epilepsy, Leigh syndrome
	Birth	Compound heterozygous c.91C>T (p.Arg31*) and c.565 T>G (p.Cys189Gly)	Developmental regression, epilepsy, apneas, Leigh syndrome, hepatomegaly, leukodystrophy, psychomotor retardation
	4 weeks	Homozygous c.1065‐3C>A	Psychomotor retardation, epilepsy, leukodystrophy
	16 months	Homozygous c.64‐2A>G	Developmental regression, myopathy, chorea, tremor, Leigh syndrome
Parfait et al. (2000)	9 months	Compound heterozygous c.1595C>T (p.Ala24Val) and c.25A>C (Met initiation codon to Leu)	Psychomotor delay, Leigh syndrome
Horvath et al. (2006)	5 months	Compound heterozygous nonsense mutation (p.Trp119*), missense mutation (p.Arg83Val)	Leigh syndrome
Pagnamenta et al. (2006)	22 months	Homozygous c.1664G>A (p.Gly555Glu)	Leigh syndrome
Alston et al. (2012)	3 months	Compound heterozygous c.1523C>T (p.Thr508Ile) and c.1526C>T (p.Ser509Leu)	Cardiomegaly, developmental delay, hypotonia, leukodystrophy
Levitas et al. (2010)	Various; 15 patients aged between 32 weeks gestation to 10 years	All 15 patients: homozygous c.1664G>A (p.Gly555Glu)	Dilated cardiomyopathy
Ma et al. (2014)	4 years	Compound, heterozygous: c.G117G/del (stop codon at residue position 56) and c.T220 T/insT stop codon at residue position 81)	Leigh‐like syndrome‐ progressive neuromuscular decline and gross motor developmental regression,
Van Coster et al. (2003)	5.5 months	Homozygous c1664G>A (p.Gly555Glu)	Unknown syndrome with hypotonia, hepatosplenomegaly, cardiomegaly, and inspiratory wheezing resulting in the death from respiratory infection
Current Proband	2 years	Compound heterozygous c.91C>T (p.Arg31*) and c.454G>A (p.Glu152Lys)	Moderate intellectual disability, ataxia, nystagmus, hypotonia, cerebellar atrophy

Counseling this family appropriately, with respect to each family member's risk of disease, was complicated as pathogenicity of the variants was inferred based on the proband's findings. Consideration had to be given to both the potential risk for tumorigenesis and of adult‐onset mitochondrial disease, such as ataxia. Individuals with known pathogenic variants in *SDHA* are recommended to receive annual biochemical and clinical surveillance for signs and symptoms of paraganglioma and pheochromocytoma syndrome (Else et al., 2008). This screening includes full‐body MRI, and blood and urine testing. There is no similar screening or testing recommended for individuals potentially at risk for adult‐onset mitochondrial disease. Additionally, the absolute risk for both conditions is unknown.

The proband's maternally inherited allele (c.91C>T (p.R31*)) has been reported in Leigh syndrome and tumorigenesis (Renkema et al., 2015), specifically PGL/PCC (Casey et al., 2017; Korpershoek et al., 2011) and gastrointestinal stromal tumor (Casey et al., 2017; Oudijk et al., 2013; Pantaleo, Astolfi, et al., 2011; Pantaleo et al., 2011; Wagner et al., 2013). Renkema et al. described this variant in a compound heterozygous state with c.565T>G in an individual with reduced complex II activity who was diagnosed with Leigh syndrome and died at 14 months of age; prior to this, the variant had only been reported in cancer syndromes (Renkema et al., 2015). Korpershoek et al. identified this variant in 0.3% of healthy control patients and 3% of sporadic tumor patients. Finally, this variant has been described in GIST when in trans with other *SDHA* variants (Korpershoek et al., 2011). Therefore, both the proband and his mother were considered at risk for developing these cancers and routine screening following the criteria specified for *SDHA* positive individuals was initiated (Lenders et al., 2014). Although the paternal allele was reported as a VUS, given the proband's positive ETC results and predictions of damaging and probably damaging by SIFT (Vaser et al., 2016) and PolyPhen‐2 (Adzhubei et al., 2010), respectively, we counseled the family as though it were a pathogenic variant and recommended the father also receive routine screening. Additional family member testing was recommended to elucidate other at‐risk individuals.

Further, given numerous reports of adult‐onset mitochondrial disease in heterozygous individuals, it is difficult to know our proband's parents’ future risk of developing neurologic symptoms. Unfortunately, while we could make them aware of this possibility, there are no actions they can take to mitigate this potential risk. Understandably, this additional information about new disease risk was overwhelming and somewhat unwelcome.

This case may represent an expansion to the phenotype associated with SDHA‐related disease and highlights a challenging counseling issue; seemingly healthy parents can be found to be at risk for conditions not previously considered and for which there are no current symptoms. The proband exhibited a disease phenotype, and had reduced ETC, and complexes II and III activity, in keeping with mitochondrial disease. The lack of symptoms in the parents suggests that the phenotype in the proband is due to biallelic loss of function of *SDHA*. This is, therefore, highly suggestive of the reported VUS, c.454G>A (p.E152K), being pathogenic and suggests that the paternal family may too be at risk of *SDHA* related conditions. The possibility of mitochondrial disease should, therefore, be considered in a child presenting with ataxia, intellectual disability, and/or developmental delay and we encourage physicians to consider *SDHA* mutations in their differential diagnosis.

## CONFLICT OF INTEREST

The authors have declares no conflicts of interest.

## AUTHORS’ CONTRIBUTIONS

BRHS designed, planned, and drafted the manuscript. EFM and EGL contributed to the writing and revising of the manuscript for important intellectual content. BRHS, EFM, PM, SK, JM, CJT, and EGL were involved in patient evaluation and management. IY performed benchwork on patient's cells for pathogenicity analysis. All authors gave the final approval of the version to be published and agreed to be accountable for all aspects of the work in ensuring that questions related to the accuracy or integrity of any part of the work are appropriately investigated and resolved.

## Data Availability

All discussed variants were submitted to ClinVar (https://www.ncbi.nlm.nih.gov/clinvar/).
